# Strain-level analysis reveals the vertical microbial transmission during the life cycle of bumblebee

**DOI:** 10.1186/s40168-021-01163-1

**Published:** 2021-11-04

**Authors:** Qinzhi Su, Qinglin Wang, Xiaohuan Mu, Hao Chen, Yujie Meng, Xue Zhang, Li Zheng, Xiaosong Hu, Yifan Zhai, Hao Zheng

**Affiliations:** 1 College of Food Science and Nutritional Engineering, China Agricultural University, Beijing, 100083 China; 2 Shandong Institute of Plant Protection, Shandong Academy of Agricultural Sciences, Jinan, 250100 China; 3 Department of Entomology, College of Plant Protection, China Agricultural University, Beijing, 100083 China

**Keywords:** *Bombus terrestris*, *Apis mellifera*, *Apis cerana*, Gut microbiota, Strain diversity, Vertical transmission, CAZyme

## Abstract

**Background:**

Microbial acquisition and development of the gut microbiota impact the establishment of a healthy host-microbes symbiosis. Compared with other animals, the eusocial bumblebees and honeybees possess a simple, recurring, and similar set of gut microbiota. However, all bee gut phylotypes have high strain-level diversity. Gut communities of different bee species are composed of host-specific groups of strains. The variable genomic regions among strains of the same species often confer critical functional differences, such as carbon source utilization, essential for the natural selection of specific strains. The annual bumblebee colony founded by solitary queens enables tracking the transmission routes of gut bacteria during development stages.

**Results:**

Here, we first showed the changes in the microbiome of individual bumblebees across their holometabolous life cycle. Some core gut bacteria persist throughout different stages of development. Gut microbiota of newly emerged workers always resembles those of their queens, suggesting a vertical transmission of strains from queens to the newborn workers. We then follow the dynamic changes in the gut community by comparing strain-level metagenomic profiles of queen-worker pairs longitudinally collected across different stages of the nest development. Species composition of both queen and worker shifts with the colony’s growth, and the queen-to-worker vertical inheritance of specific strains was identified. Finally, comparative metagenome analysis showed clear host-specificity for microbes across different bee hosts. Species from honeybees often possess a higher level of strain variation, and they also exhibited more complex gene repertoires linked to polysaccharide digestion. Our results demonstrate bacterial transmission events in bumblebee, highlighting the role of social interactions in driving the microbiota composition.

**Conclusions:**

By the community-wide metagenomic analysis based on the custom genomic database of bee gut bacteria, we reveal strain transmission events at high resolution and the dynamic changes in community structure along with the colony development. The social annual life cycle of bumblebees is key for the acquisition and development of the gut microbiota. Further studies using the bumblebee model will advance our understanding of the microbiome transmission and the underlying mechanisms, such as strain competition and niche selection.

Video Abstract

**Supplementary Information:**

The online version contains supplementary material available at 10.1186/s40168-021-01163-1.

## Background

Animals are colonized by a myriad of microbes that play important roles in host health. In many cases, the symbioses are perpetuated by the transmission of symbionts through host generations and play an important role in host evolution [1]. The associated microbes can be transmitted both horizontally between individuals by sharing environment or interpersonal interactions and vertically from the mother to the progeny [2]. Increasing evidences have shown that microbial associations are present in diverse animals and are functionally essential. Social insect symbionts are also inherited via vertical transmission [3]. Previous studies have shown that newly emerged bumblebees and honeybees have few microbes, suggesting that microbes do not pass through metamorphosis [4]. However, how social transmission and metamorphosis affect the acquisition process of vertically transmitted microbes remains elusive. This is mainly due to the difficulty to track the changes and potential exchanges of diverse microbiota across hosts.

Compared with other animals, social bumblebees (*Bombus* spp.) and honeybees (*Apis* spp.) harbor a simple, recurring, and similar set of gut microbiota, including shared core phylotypes of *Gilliamella*, *Snodgrassella*, *Bifidobacterium*, *Lactobacillus* Firm-4 and Firm-5, and several host-specific phylotypes (e.g., *Frischella* for *Apis*; *Schmidhempelia* for *Bombus*) [5]. Although the bee guts consist of a limited number of bacteria phylotypes, metagenomic analysis showed that the bacterial communities exhibit substantial strain-level diversity in honeybee guts [6, 7]. Interestingly, for the eusocial honeybees and bumblebees, their microbiota is stably maintained through social contacts. However, they differ strongly in the life cycle and social behaviors. Honeybee colonies usually are larger and are initiated by swarming, and the bacterial pools are carried by thousands of worker bees and can be transmitted to future generations. Through metamorphosis, honeybees are associated with different kinds of microbes as larvae and adults. The larval gut compositions are always erratic, with bacterial species less prevalent in the adult guts, which are acquired from the diet [8]. Newly emerged honeybees are microbiota-depleted, and a stable gut community establishes during the following days after eclosion. Experiments with newborn, microbiota-free honeybees showed that exposure to hive materials and social contact with nestmates is essential for establishing a typical gut composition [9]. In contrast, the colony cycle of bumblebee is annual, not perennial as in honeybees. Bumblebee colonies are founded by single-mated queens, who take charge of the foraging, nest-building, and nursing activities in newly established nests. Thus, it is reasonable to hypothesize that the founding queen must be the sole carrier of gut symbionts and transmit them to the next generation. Based on these characteristics of bumblebees, they serve as a more suitable model to track microbiota transmission routes, specifically the contributions of behaviors and social interactions in shaping microbiota compositions.

Although the structure and characteristics of the gut microbiota of honeybees have been well studied, few examinations focused on the bumblebee gut. In particular, all observations are based on 16S rRNA gene analysis, which could not resolve the genomic level variations in strains within phylotypes [7]. Examinations based on single-copy genes of the bee gut symbiont *Snodgrassella alvi* demonstrate that specific set of strains are associated with *Bombus* hosts [5]. Furthermore, strain-level variations exist across and within individuals of bumblebees, which has also been observed in honeybees by both amplicon and shotgun sequencing [6, 10]. Interestingly, by deep sequencing of the single-copy coding gene (*minD*), it revealed that strain diversity is much lower in *Bombus* than that in *Apis* [10], suggesting that the life cycle and colony development affect strain transmission across generations. Genomic data of the isolates have shown that strain-level variants within microbial species are essential in determining functional capacities, specifically in diet polysaccharide metabolism [11, 12]. Therefore, it is crucial to identify and assess the transmission of strains with distinct genetic repertoires, which probably affect the establishment of a healthy symbiosis and host biology [13]. Koch et al. showed that daughter queens acquire two core gut members from the mother colony, facilitated by social contact after pupal emergence [14]. However, 16S rRNA-based analysis was not able to track specific genotypes for some of the stains overlapping the daughters and the maternal source [14]. Strain-level metagenomic profiling is needed to infer the transmission at higher taxonomic resolutions.

In this study, we firstly profiled the microbiome structures associated with bumblebees (*Bombus terrestris*) from different life stages. As found in honeybees, metamorphosis primarily affected microbial compositions in bumblebees. However, some core bacterial species were already present in eggs, and certain strains persist in lateral life stages, suggesting a vertical transmission of bacteria. By examining both diapause and ovipositing queens as well as their worker gut samples, we assessed the relatedness of taxonomic profiles within colonies. To analyze species- and strain-level transmission events, we described the structure of gut communities using whole-genome metagenomic sequencing. A longitudinal study was designed to follow the dynamic changes of paired queen-worker samples at different development stages of the colony. Based on a custom bacterial genomic database and survey of the single-nucleotide variant (SNV) patterns, we characterized the vertical inheritance of strains between queen and worker. Finally, we compared the species composition and functional profiles of the gut microbiota of both bumblebee and honeybee hosts. Our results further document the host specificity of strains and the strain-level diversity of bacterial species, which is linked to the different life cycles of hosts.

## Methods

### Sampling of bumblebee from different stages of development

**Fig. 1:**
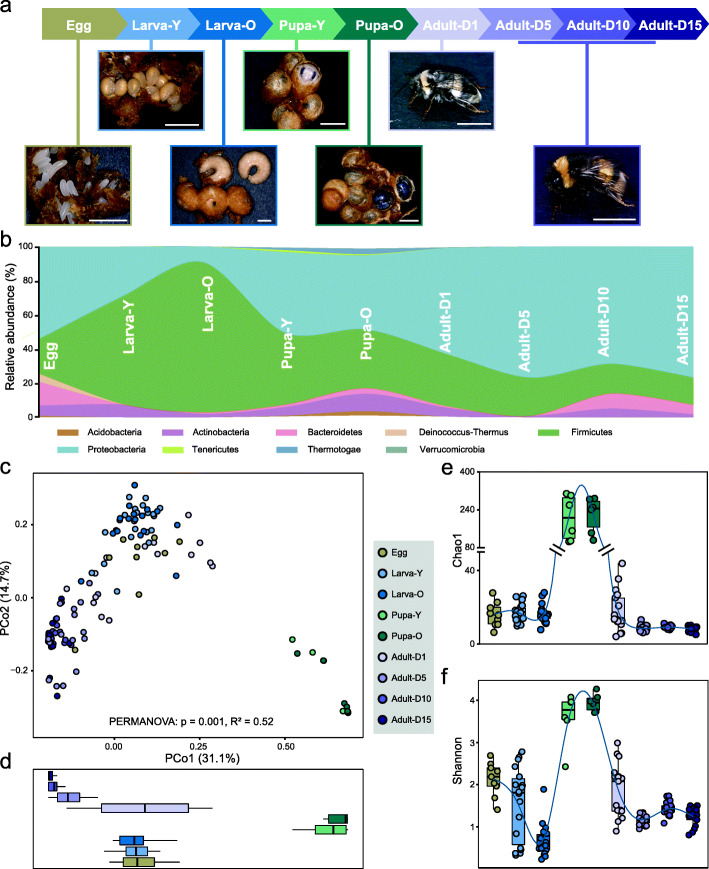
Microbiota compositions of bumblebee shift along the developmental stages. (**a**) Samples were collected from different stages of bumblebee development. Clusters of eggs and young larvae (Larva-Y) living together were collected from the brood clumps. Old larvae (Larva-O) spin individual strong silken cocoons and no longer continued to feed. Young and old pupae (Pupa-Y and Pupa-O) were dissected from closed cells. Adult bees were sampled 1, 5, 10, and 15 days after the eclosion (Adult-D1, -D5, -D10, D15). The hairs of 1-day-old bees are entirely white, and the characteristic coloration started to develop in about 24 h. (**b**) Relative abundance of phyla shifts during the developmental stages as shown in a stream graph. (**c**) Microbiome compositions in different developmental stages plotted on an unweighted UniFrac PCoA graph. (**d**) Boxplots indicate the distribution of each life stage along the first principal coordinate (PCo1). (**e**–**f**) Chao1 (**e**) and Shannon (**f**) diversity metrics of the data sets. The blue fit lines were obtained by using a generalized additive model (GAM)

Original colonies of *B. terrestris* were purchased from Bioforce Ltd. Karaka (Papakura, New Zealand) and then were reared year-round in a climate-controlled room at 28 °C, 65% relative humidity, and continuous darkness. Bees were fed on sterilized pollen and sugar syrup (50% sucrose solution, w/v) ad libitum. We collected bumblebees from nine age periods from established colonies in the nest-box (15 × 20 × 20 cm; Fig. 1a). Eggs were ~2-day old, and 3–6 egg cells from the same nest were pooled to enable the extraction of enough DNA. We defined the ~4-day old non-isolated larvae (2nd instar) living within the same brood clump as “Young Larvae.” The isolated 4th instar larvae collected from separate sealed cocoons were defined as “Old Larvae.” Since the developmental timing of pupae varied for different sizes of bees, we sampled pupae with similar sizes from two stages, respectively. Pupal stages were defined as described by Tian et al. [15]. “Young Pupae” (P7 stage) with white body and pigmented eyes were dissected from the cocoons ~4 days after pupation. “Old Pupae” (P13–P14 stage) with black abdomen and head were sampled ~6 days after pupation. To obtain age-controlled adults, newly emerged bees within a 12-h period were paint marked and re-introduced to their nests. They were re-collected on day 1, 5, 10, and 15 after eclosion.

To follow the dynamic changes in the gut community during the development of nests, we set up 15 independent colonies simultaneously. Firstly, toward the end of the colony cycle, newly emerged males and queens were separately placed in cages. Single-time mating was performed with queens and drones from different nests and mated queens were fed on sugar syrup and pollens for ~7 days before the artificial hibernation at 3 °C for 12 weeks. We placed 132 queens who emerged from hibernation individually into small boxes (7.5 × 5 × 9 cm) with two ~15-day old workers for the nest initiation. At the same time, 18 diapause queens were sampled. One month later, the queens laid eggs actively and were moved to bigger boxes (15 × 20 × 20 cm) together with the eggs and nest materials, but without the founding workers. Then, we sampled queens and workers from three development stages of the nest (Fig. 4a). Once the first batch of workers (3–5 individuals) emerged, newly emerged workers were sampled with their queens (“New” stage). The first batch of workers of the remaining nests was labeled with paint on top of the thorax. After 15 days, they were sampled together with their queens, and this stage was defined as “Early.” After ~1 month, the remaining nests fully developed with ~50 workers, we started to label a batch of newly emerged workers. When this batch of workers was 15 days old, we sampled them with their queens, and this period was defined as “Late.”

### qPCR assay

The whole specimens of pooled eggs and larvae were surface sterilized using 75% ethanol. Whole guts of pupae and adults were dissected using sterile forceps. DNA of all samples was extracted using CTAB method as previously described [16]. Absolute abundance of the bacterial community was determined by quantitative PCR. All qPCR reactions were carried out on the QuantStudio 1 real-time PCR system (Thermo Fisher Scientific, Waltham, USA) with universal bacteria primers [17]. Standards for target genes cloned into the pCE2 TA/Blunt-Zero Vector (Vazyme Biotech, Nanjing, China) were created by PCR amplification of the genomic DNA of *Gilliamella apicola* strain W8127. Each reaction was performed in triplicates on the same plate in a total volume of 10 μl (0.2 μM of each forward and reverse primer; ChamQ Universal SYBR qPCR Master Mix, Vazyme Biotech) with 1 μl of DNA.

### 16S rRNA gene sequencing and processing

Targeted amplicons of the V4 region of 16S rRNA gene were generated with primers 515F and 806R [18]. Sequencing libraries were generated with NEBNext Ultra II DNA Library Prep Kit for Illumina (New England Biolabs, Ipswich, USA) and were sequenced on the Illumina NovaSeq 6000 platform (2 × 250 bp). DNase free molecular grade water and the genomic DNA of *Escherichia coli* were used as negative and positive controls to test the quality during the library preparation. Bioinformatic analysis was implemented using QIIME2 pipeline [19]. Briefly, quality control, chimera checking, and pair-end read merge were processed with DADA2 [20]. Taxonomy was assigned to representative sequences with the q2-feature-classifier plugin [21] using the curated database for bumble and honeybee gut microbiota [22]. We then generated the BIOM file containing information on the read counts and taxonomy of features (ASVs) using QIIME2. MicrobiomeAnalyst platform was used for the community profiling and statistical analysis [23].

### Metagenomic shotgun sequencing and binning

Sequencing libraries for shotgun metagenomics were prepared with the NEBNext Ultra II DNA Library Prep Kit for Illumina and were sequenced on the Illumina NovaSeq 6000 platform (2 × 150 bp). DNase-free molecular grade water and the genomic DNA of *Escherichia coli* were used as negative and positive controls to test the quality during the library preparation. Adaptor trimming and quality control of the raw sequencing data were carried out using fastp [24]. Then, clean reads of each metagenomic sample were first mapped against the reference genome of *B. terrestris* host (GCA_000214255) using BMTagger (ftp://ftp.ncbi. nlm.nih.gov/pub/agarwala/bmtagger/) to filter off host-derived read. We also processed 76 published metagenomes from honeybee gut samples following the same pipeline as described above [6, 7], and the genomes of *A. mellifera* (GCA_003254395) and *A. cerana* (GCA_001442555) were used as reference accordingly.

*De novo* metagenomic assembly was carried out for both reads from individual gut samples (single-sample assembly) and pooled samples from the same nest (co-assembly) with metaSPAdes [25]. Metagenomic binning was performed using the metaWRAP pipeline [26]. The completeness and contamination of each bin were estimated using CheckM [27]. After dereplication, a total of 28 metagenome-assembly genomes (MAGs) were obtained (> 75% completeness, < 10% contamination). Each bin was taxonomically assigned against the Genome Taxonomy Database with GTDB-tk [28] (Additional file 1: Supplementary Table S1).

### Species- and strain-level community profiling

The species- and strain-level community profiling and gene content estimation were performed using the Metagenomic Intra-Species Diversity Analysis System (MIDAS) pipeline [29]. A custom bee gut microbial genomic database was generated based on 449 isolate genomes and 28 MAGs from this study (Additional file 1: Supplementary Table S1). Pairwise genomic average nucleotide identities (gANI) for strains within each phylotype were calculated with fastANI [30]. For genomes from each phylotype, a set of 120 ubiquitous Bacteria domain-specific single-copy marker genes as being present in > 90% of bacterial genomes in GTDB database [31] was identified, aligned, and concatenated into a single sequence alignment by GTDB-tk [28]. A maximum-likelihood tree was inferred with FastTree based on the amino acid sequences [32]. Following the instruction of MIDAS, strains from each phylotype showing a minimum pairwise gANI of 95%, a gold standard definition of prokaryotic species, and forming a monophyletic clade with 1.00 SH-like support values were defined as one species cluster [29]. Based on the whole genome phylogeny, all strains were classified into 86 bacterial species. To estimate the relative abundance of species clusters, quality-filtered reads from metagenomes were mapped with the “run_midas.py species” module. Then “merge_midas.py species” was used to combine results across all samples.

The strain-level profiling was performed by identifying SNV diversity for each species cluster in metagenomic samples using the “run_midas.py snps” module. Representative genomes with the highest completeness and lowest contamination were selected for each species cluster (Additional file 1: Supplementary Table S1). We ran “run_midas.py snps” on the metagenomes to compute the allele frequency per-sample-per-species cluster along the entire reference genomes. Then “merge_midas.py snps” function identified the bi-allelic SNVs prevalent in more than 5% of samples and removed rare SNVs with abnormally high ratio of site depth to genome depth (--site_ratio FLOAT = 2.0). Strain-level diversity within species clusters in each sample was estimated by quantifying the fraction of SNVs in protein-coding genes (number of SNVs/length of genes). To identify the phylogeny of the strain profiles of individual samples, we used the script “call_consensus.py” to generate core genome consensus alignment with variable sites in CDS. The phylogenetic tree was reconstructed by FastTree with the maximum likelihood method.

### Functional profiling of metagenomes

To compare the functional diversity among metagenomic samples, we used the “Metagenomic pan-genome profiling” module of the MIDAS pipeline. Firstly, protein-coding regions of all genomes from the database were predicted using Prokka [33], and the amino acid sequences were annotated against the Kofam database using KofamKOALA [34]. KOs were assigned to each amino acid sequence according to adaptive score thresholds. Bacterial genomes were also annotated using dbCAN2 [35]. A 99% sequence identity threshold was used to generate distinct gene clusters, and a centroid gene sequence was identified from each cluster by VSEARCH [36]. Shotgun reads were mapped against the centroids using the “run_midas.py genes,” and mapped reads were used to compute the coverage of genes. Gene coverages were normalized by the median coverage across a set of 15 universal single-copy gene families with “merge_midas.py genes.” Then, the copy number of genes was multiplied by the relative abundance of each species cluster. The abundance of each KO or CAZyme family in the metagenomic samples was obtained by summing over all genes annotated with the same KO or CAZyme annotation.

## Results

### Microbiome composition shifts during developmental stages

We collected 127 bumblebee samples from four distinct metamorphosis phases: egg, larva, pupa, and adult. Following the gut composition across different life stages, we found that the 16S rRNA amplicon profiles at phylum-level dramatically changed with the development (Fig. 1b). PCoA showed that the microbiomes of individual bumblebees, when plotted by life stages, formed separate clusters (Fig. 1c, top panel). Notably, the gut composition of the two pupa groups was isolated from the other stages (Fig. 1c and d), and they possess very few gut bacterial cells (Additional file 2: Supplementary Fig. S2). The microbiome of eggs and larvae are relatively closer, and the gut community of newborn adults (Adult-D1) is segregated from the other adult age groups. Accordingly, the guts of larvae possessed extraordinarily higher richness and were composed of more diverse community members (Fig. 1e and f). With the development from egg to larval stage, the diversity of the associated community decreased (Fig. 1), and the values of Chao1 and Shannon index are both moderately higher in 1-day-old adults and were stable in the older bees.

**Fig. 2:**
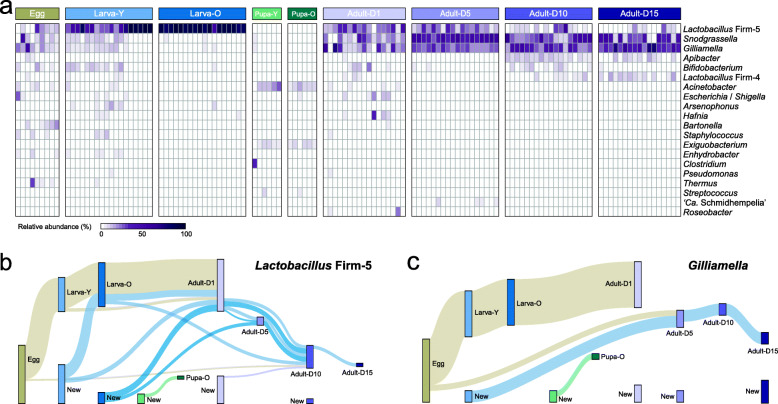
Major taxa and OTUs transferred during the development. (**a**) Heatmap shows the dominant taxa from all individuals sampled across the developmental stages. (**b**–**c**) Amplicon sequence variants (ASVs) in both the phylotype of *Lactobacillus* Firm-5 (**b**) and *Gilliamella* (**c**) present across developmental stages are tracked using Sankey plots. The heights of the rectangles indicate the relative number of ASVs, and colors distinguish each developmental stage. The shades represent the transfer of ASVs between stages, and their colors indicate the first stage of appearance

The distribution and abundance of bacterial phylotypes in different life stages agreed with the overall clustering observed in the ordination analyses (Fig. 2a). Previously identified core gut members of bumblebees (i.e., *Lactobacillus* Firm-5, *Snodgrassella*, *Gilliamella*) were present in all developmental stages, except in pupae. Although several lineages of bacteria such as *Snodgrassella*, *Gilliamella*, and *Bifidobacterium* were present in young larvae, the guts of old larvae were exclusively dominated by *Lactobacillus* Firm-5 (Fig. 2a; Additional file 2: Supplementary Fig. S3). As observed in the ordination analysis, the communities of pupae were distinct from all other stages, and only several genera probably acquired from pollen fed to the colonies (e.g., *Acinetobacter* [37]) were detected. After eclosion, the adult guts were mainly composed of the core members, and the relative abundance of *Gilliamella* and *Snodgrassella* increased with the growth of adults. In addition, *Apibacter* that is specifically prevalent in *Bombus* spp. and *A. cerana* was only abundant in 10- and 15-day old adults (Additional file 2: Supplementary Fig. S3). These results indicated that the community members are volatile with the development of bumblebees, which correlated with their complete metamorphosis.

While most of the core members were demised during the pupal stages, some bacterial lineages were prevalent through the development, specifically members of *Lactobacillus* Firm-5 and *Gilliamella* were observed in eggs, larvae, and adults. Thus, tracking the dynamics of individual ASVs within phylotypes across the development provides an opportunity to follow the transmission process. Many of the *Lactobacillus* Firm-5 ASVs were stable in eggs, larvae, and newborn adults; however, they disappeared in older adult guts (Fig. 2b). Several Firm-5 ASVs originating from the young and/or old larval stage (blue shades) reappear in different age groups of adults. ASVs of *Gilliamella* showed a similar volatile pattern (Fig. 2c). Many ASVs of *Gilliamella* were shared across eggs, larvae, and the newly emerged adults (beige shades), but these strains were lost in the guts of older workers. Although new members were always identified in the adult guts, ASVs detected in the egg and young larval stages reappeared in older adults (> D5), which indicates that some strains are stably inherited between individuals from different development stages. In contrast, strains from both phylotypes detected in pupae were all exclusive to this stage (Fig. 2b and c).

### Newborn workers consistently resemble their queens in microbial community

**Fig. 3:**
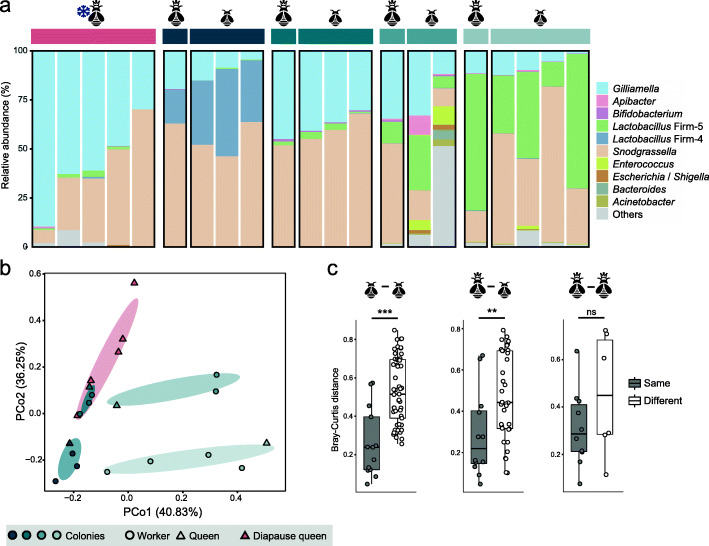
Gut microbiota compositions of the queen and worker bumblebees during hibernation and early stage of nest establishment. (**a**) Relative phylotype-level abundance profiles for the queen and newly emerged worker samples. Queen-worker paired samples were simultaneously collected from four independent nests. (**b**) PCoA plot based on the Bray-Curtis distance between samples highlights the spatial clustering of samples concerning the queen-worker pairs. (**c**) Gut compositions are more similar for bees from the same nest as shown by Bray-Curtis dissimilarity of individual workers (left), queen-worker pairs (center), and queens (right). ***p < 0.001; **p < 0.01; ns, not significant (Wilcoxon test)

To elucidate the nature of queen-worker microbiota transmission in the early stage of nest establishment, we enrolled four replicating newly established nests. Gut samples were simultaneously collected from the first batch of newborn workers of the nests and the maternal samples. Meanwhile, we sampled guts from the same batch of diapause queens. We first characterized the relative abundance profiles of bacterial genera, and all guts were dominated by the core bee gut members (Fig. 3a). However, the community compositions were distinctive for different nests, indicating that the microbiota is variable for the initiating colonies. Nevertheless, all diapause queen communities had a high fraction of *Gilliamella* and *Snodgrassella*. Clustering based on the ASV-level profiles revealed that samples from the same nest were more closely related (*R*^2^ = 0.64, *p* = 0.001; PERMANOVA), and diapause queen samples formed a separate cluster (Fig. 3b).

Interestingly, although the gut communities are variable among nests, the gut composition of workers resembles their maternal samples. To compare the queen and newborn worker microbial community, we examined the variability of the gut compositions within and across different nests. By testing the ASV-level Bray-Curtis dissimilarity, we found that the gut communities are more similar for workers from the same nest (Fig. 3). The paired queen-worker samples from the same nests also exhibited significantly similar features (Fig. 3c). The comparisons were not significantly different for queens within the diapause group and across different nests, which might be underpowered as only few queen samples were included here. These results indicate that the microbiota of newly emerged worker bees resembles that of their queens, suggesting the queen microbial reservoir as an important source in early acquisition of microbiota in the worker bumblebee gut.

### Queen gut microbiota is the major source of transmitted strains to next generations

**Fig. 4:**
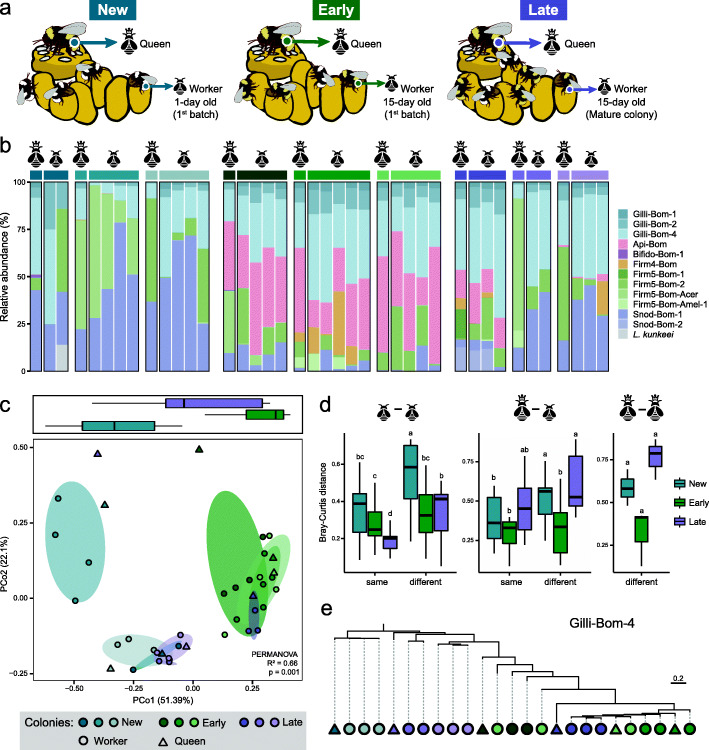
Longitudinal metagenomic sequencing of the microbiome of queen-worker pairs along with the nest development. (**a**) We sampled queen-worker paired samples from nests belonging to three age periods: newly established nests (New) with egg-laying queens and their first batch of emerged workers (1-day old); early stage of nests were sampled when the first batch of color-labeled workers were 15-day old; late stage of nests are about 45-day old since the foundation of the nest, and then a batch of newly born workers were labeled and collected after 15 days. (**b**) Bar plots showing the relative abundance of the species clusters in paired metagenomic samples from three replicate nests. (**c**) Bray-Curtis dissimilarity PCoA based on the gut community composition described at the species cluster level. Boxplots (bottom panel) show the distribution of each stage of nest development along the first principal coordinate (PCo1). (**d**) Bray-Curtis dissimilarity of species cluster-level composition profiles of worker-worker, queen-worker, and queen-queen paired samples from the same or different nests over time. (**e**) Strain-level phylogenomic tree of the “Gilli-Bom-4” species cluster based on the concatenated alignments of consensus-alleles found in the core-genome

Thus far, we showed that for the very first batch of workers, the major source of microbiota is provided by the queen who initiated the nest. However, as the nest development progresses, queens undergo transitions in maternal care behavior [38]. The workers take over most brood-feeding activity on further batches of their siblings, and queens specialize in reproduction. Therefore, the transmission pattern of gut symbionts may shift with the nest growth. We designed a paired longitudinal study and followed the gut composition of the queen and worker samples with the nest development. We initiated replicating colonies and collected queen samples together with 2–5 age-controlled worker samples at different periods: at emergence of the first batch of workers, 15 days after emergence, and from mature colonies with ~50 workers (Fig. 4a). Moreover, to analyze the species- and strain-level composition of the microbial communities, we performed high-resolution shotgun metagenomics for all 40 samples in this cohort (Fig. 4b).

First, we built a genomic database based on bee gut isolates and MAGs obtained here for the comparative analysis of shotgun metagenomes. Phylogenomic analysis showed distinct clusters for most isolates from different host species (Additional file 2: Supplementary Fig. S1), which agrees with previous genomic analysis [11, 12]. Quantitative taxonomic profiling revealed that the relative abundance patterns of bacterial species shift with the development of the nest. Only one species cluster of *Apibacter* specific to *Bombus* was identified in our cohort, and its abundance is significantly higher during the early stage (Additional file 2: Supplementary Fig. S4a). With the development of the nest, the abundance of *Gilliamella* increased, whereas *Snodgrassella* dramatically dropped in early and late stages. We then characterized the gut structure at the level of species. First, only *Bombus*-specific species were present in the guts, while two clusters from *Lactobacillus* Firm-5 contained genomes from both *B. terrestris* and *Apis* spp. hosts. As observed in honeybee gut [6, 7], multiple species clusters from the phylotype co-exist in the guts of individual bumblebees. Although we defined 12 species clusters for *Gilliamella* isolates from bumblebees in our database, only three clusters were identified in our cohort. Notably, all samples were dominated by Gilli-Bom-4, which also possessed the most isolate genomes and MAGs in the database. In contrast, only one species of *Lactobacillus* Firm-4, *Bifidobacterium*, and *Apibacter* were present in our samples, respectively (Fig. 4b). Unlike *Snodgrassella* from *A. mellifera* and *A. cerana* with only one species cluster, strains from *Bombus* spp. could be clustered into five species (Additional file 2: Supplementary Fig. S1b). However, in our cohort, Snod-Bom-1 was dominant, and only one queen and two workers from one late stage of colony had more proportion of Snod-Bom-2 cluster.

To test whether the gut microbiota changed during the development of the nest, we conducted PCoA based on the species-level profiles. Clustering according to the nest development stage revealed that gut structures of bees from the same colony were more closely related (Fig. 4c). To compare queen and worker gut microbiota, we examined the variability of gut communities within and across nests during the development. By testing the species-level dissimilarity, we found that the gut structures became more stable for workers from the same colony. The distances were consistently higher for individuals from different nests, indicating nest segregation (Fig. 4d). Nevertheless, the distance was significantly lower between queens and workers from the same nests at the “New” stage (Fig. 4d), which agrees with the amplicon sequencing analysis (Fig. 3). To further support intra-pair vertical transmission events, we intended to identify strain variants in the queen and their workers. We identified strain-level variations between queens and workers by mapping reads against reference genomes and obtained a survey of SNV patterns across samples. For the most prevalent cluster, Gilli-Bom-4, strain-level population structure inferred by the phylogeny for the concatenated genomes identified common strains in individuals from the same nests and stages (Fig. 4e). Moreover, they displayed clear maternal routes of transmission, which is confirmed by closely related SNV identity patterns of dominant strains between workers and their queens. Conversely, strains within the *Apibacter* species “Api-Bom” did not segregate for different nests, which is likely due to the low intra-species variation (Additional file 2: Supplementary Fig. S5).

### Bumblebee and honeybee differ in gut community composition and carbohydrate-digestive capacity

**Fig. 5:**
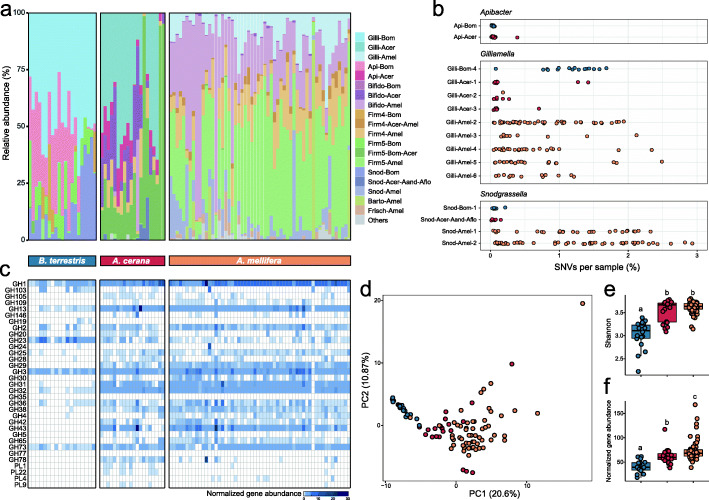
Compositional and functional profiles of the gut microbiota are distinguishable between bumble and honeybee species. (**a**) Gut microbiota of bumble (*B. terrestris*) and honeybee (*A. cerana* and *A. mellifera*) species are composed of host-specific species clusters. (**b**) Species clusters from bumble and honeybee guts exhibit a variable level of strain diversity as calculated by the proportion of SNVs of protein-coding genes within each metagenome. (**c**) The distribution of CAZyme family across bumble and honeybee gut metagenomes. (**d**) The CAZyome profiles are differentiable between bee species. (**e**–**f**) Shannon diversity index (**e**) and the abundance (**f**) of the genes encoding CAZyme in the gut metagenomes of bumble and honeybee species

Although host-specialized bacteria species were respectively detected in bumble and honeybees [10], species-level variation in gut communities across host species is yet uncharacterized. We compared the profiles of 21 shotgun metagenomes of 15-day-old bumblebee samples together with 76 previously published metagenomes of two honeybee species (20 samples from *A. cerana* and 56 samples from *A. mellifera*) [6, 7]. Overall, all three host species were dominated by the five core phylotypes, which are acquired by the common ancestor of the eusocial corbiculate bees [5]. However, the proportion of *Gilliamella*, *Apibacter*, and *Snodgrassella* in *B. terrestris* is relatively higher than in the two species of *Apis* (Fig. 5a). Two phylotypes, *Frischella* and *Bartonella*, were only detected in *A. mellifera*, and *Apibacter* was only prevalent among *B. terrestris* and *A. cerana* samples. The majority of species clusters exhibited host specificity, which agrees with previous findings by amplicon sequencing [5]. In contrast, two species clusters represented by bacterial genomes from different bee species (Firm4-Acer-Amel; Firm5-Bom-Acer) might be shared by the hosts, but they were only detected in small amounts or few individuals.

It has been suggested that the strain-level diversities are higher for species from *A. mellifera* than those from *A. cerana* [7]. Here, we compared the length-normalized rate of SNVs across the whole genomes for species clusters from *Apibacter*, *Gilliamella*, and *Snodgrassella*, which are present in both *B. terrestris* and *Apis* species. Both species clusters from *Apibacter* specific to *Bombus* and *A. cerana* showed a low level of strain variation (Fig. 5b). For *Gilliamella*, Gilli-Bom-4 specific to *Bombus* had > 1% SNVs in most samples, while the diversity is quite variable in honeybee individuals. Conversely, the variety of two *Snodgrassella* species from *Bombus* and *A. cerana* were generally genetically consistent, and *A. mellifera* exhibited high inter-sample variation. These results indicated that strain-level diversities varied in species clusters from different phylotypes and different host species.

The differences in species and strain composition may result in distinct functional profiles among gut communities of the three host species. We then calculated the abundance of genes encoding CAZymes by mapping metagenomic reads to the genome database, which had been annotated against the KO and dbCAN2 databases. The relative abundance pattern of the KEGG pathways was indistinguishable among three different hosts, and the pathways of carbohydrate metabolism, amino acid metabolism, and membrane transport were abundant in the samples (Additional file 2: Supplementary Fig. S6). In contrast, the gut microbiota exhibited distinct repertoires of CAZymes. Both honeybee hosts harbored more contents of GH and PL genes compared to *B. terrestris*, while only the GH23 family was enriched in bumblebee individuals (Fig. 5c; Additional file 2: Supplementary Fig. S7). Moreover, GH43 enriched in *Bifidobacterium* isolates from *Apis* [11] was only detected in honeybee guts, corresponding to the low level of *Bifidobacterium* in the bumblebees. To compare the global CAZyme profiles, we quantified the diversity of all CAZyme genes among the three bee species. PCA indicated the distinct composition of CAZymes constituting the guts of the three bee hosts (Fig. 5d). Accordingly, *Bombus* microbiota showed less functional capacity and gene diversity for the utilization of polysaccharides than that of *Apis* (Fig. 5e and f). *Apis mellifera* possessed greater polysaccharide-utilization capacity than *A. cerana* (Fig. 5f), as expected based on a previous study [7]. Thus, these results showed that the diversity and abundance of polysaccharide-utilization genes are substantially higher in *Apis* than those in *B. terrestris*, while the general functional profiles are quite similar.

## Discussion

Although the core gut microbiota of bumblebees shares the same set of core bacterial taxa with that of honeybees [5], the social nesting behaviors of these two corbiculate bees differ radically in form. Honeybee colonies are founded by one queen together with tens of thousands of workers, and the life of colonies is always perennial. The microbiota transmission is accomplished by trophallaxis and other social contacts across generations overlapping within a shared nest [8]. In contrast, bumblebee colony is annual and is initiated by a single overwintered queen [10, 39]. Thus, the microbiota must be transmitted from the maternal queen to their progeny for generational inheritance. We found that the gut compositions differ among founding queen individuals, though queen and worker microbial communities were often significantly stable within hives, and they resemble one another (Fig. 3). These results indicate that maternal queens directly shape the microbial composition of the gut in neonate workers. Notably, there were striking differences between diapause and founding queen gut microbial community compositions. The shifts in gut community of bumblebee queens from different life stages have been shown in another species, *Bombus lantschouensis* [40]. Our results further document that the gut microbiota is associated with the physiology and life stages of the host.

Since bumblebee colony is founded by a single queen, it is easy to hypothesize that the microbiota of the newly established colony must be acquired from the queen. However, with the development of the nest, the social tasks will change for the queens. The first batch of offspring is all fed by queens, whereas after the emergence of the first batch of workers, they undertake the foraging and caring for further batch of brood. The queen focuses on reproduction and is fed on diets provisioned by workers [41]. Here, we followed the early acquisition and development of the worker and queen gut microbiota by a longitudinal metagenomic approach. Although there is high heterogeneity in the first batch of worker gut microbiota either within or across different colonies, it dramatically decreases within the following 2 weeks (Fig. 4d). This suggests a homogeneous selection force for workers with a different inoculation to maintain a recurring set of bacterial phylotypes in individuals even from different nests. This selection process is also observed in the pioneering human infant gut microbiome for the strict anaerobes, which is corroborated by the biochemical changes of the gut environment [42].

Interestingly, the gut composition of queens also changes with the nest growth, and they often reflect that of their workers (Fig. 4c). Conversely, the honeybee queen gut community is dramatically different from those of workers who feed them, and most of the core gut members of workers are absent [43, 44]. This is highly likely due to their special diet of royal jelly, which also alters the genome-wide methylation patterns during the development [45]. As workers emerge and take over the duty, bumblebee queen restricts to egg laying and feeds herself from honeypots provided by workers [41]. Thus, the diet of bumblebee queen and workers within the hive do not differ. The microbiota may exchange across all individuals within the colony through the shared honeypots and other social contacts [39, 46].

Although bumblebee nests are initiated by single queens, which may impose a bottleneck on the generational inheritance of the gut microbiota [5], they maintain associations with a host-specific and recurring set of microbiota. We have documented that the founding queens are the primary carrier of the microbiota for newly established colonies; however, metamorphosis with divergence in physiology between different development stages causes constraints on the transmission of symbionts [47]. While the microbial composition varies during the metamorphosis (Fig. 1), some core members, such as *Lactobacillus* Firm-5 and *Snodgrassella*, are dominant during the larval and egg stage of bumblebees (Fig. 2). In contrast, honeybee larvae are exclusively composed of *Parasaccharibacter apium* (Alpha 2.2) prevalent in the hypopharyngeal glands and royal jelly of honeybees [48, 49]. Larvae of the pollen-storer *B. terrestris* feed on a liquid mixture of pollen and honey without secretions from the adult workers [50, 51]. In contrast, honeybee larvae continuously consume hypopharyngeal gland secretions from the nurse adults [52]. We find that pupae possess a much lower level of bacteria than the other stages, and only non-core species are detected (Fig. 2a and S2). This is quite similar to the shift in gut microbiota during the development of honeybees [8, 48, 53]. The guts of newly eclosed bumblebees are already colonized with core bacterial members, and the absolute abundance quickly increases within few days postemergence. During metamorphosis of insects, the entire gut is reorganized and almost all of the gut contents are eliminated. This may cause gut symbionts to be lost entirely [47]. Facing the constraints on symbiont maintenance, eusocial insects transmit symbionts among adult nestmates [3]. Specifically, newborn honeybees acquire core gut bacteria through social contacts and sharing the hive environment with nestmates [9]. Unlike honeybees, bumblebees do defecate in and close to the nest [38], but they have no trophallaxis (oral food exchange) [54]. Although microbiota-free bumblebees can be generated by pulling out pupae from the brood cells [55], newly emerged bumblebees not raised in a sterile environment here already possessed gut bacteria. Thus, sociality, including sharing food stores and hive environments (e.g., cocoon material and feces), must be the primary route for the transmission of microbiota for bumblebees [56].

Interestingly, a low amount of bacteria were detected in the eggs, but they are composed of core gut members of the bumblebee microbiota (Fig. 2a). This indicates that these phylotypes may be present in the female reproductive tract and vertically transmitted from during egg-laying. Alternatively, *B. terrestris* are progressive provisioners (pollen storers), and workers regularly provide the larvae with food in progressive provision [38]. Therefore, microbes can be transmitted to the eggs laid on the pollen clump and wax layer mixed with pollen. Moreover, the identification of the shared ASVs across developmental stages indicates that a substantial fraction of the strains from *Lactobacillus* Firm-5 and *Gilliamella* are transmitted from eggs to larvae (Fig. 2b). Common strains are also found between larvae and older adults (> 5 days), demonstrating nursing behavior as a significant route for the transmission of larva-acquired strains. However, 1-day-old adults have ASVs that do not persist in the older bees, suggesting a selection process for niche-specific stains from the microbial pools as found in the colonization of microbiota in early childhood [42]. Furthermore, our metagenomic data allowed the assessment of strain-level diversity for core species prevalent in the population simultaneously. Strain-level phylogeny based on SNV identity patterns showed that the worker samples possess closely related strains with their queens, indicating a clear maternal route of transmission (Fig. 4). Moreover, strains of the “Gilli-Bom-4” species form distinct groups for different nests, suggesting each colony possesses a specific set of strains. Compared to the colony production by swarming for honeybees, the retention of strain diversity depends mainly on the founding queen herself of *Bombus* species. Remarkably, this bottleneck effect leads to a lower genetic diversity of strains in bumblebees [10], while the extent of genetic divergence of strains within species varied significantly for different gut members. For example, the SNV rates were extremely low for the “Api-Bom” and “Snod-Bom-1” species clusters, corroborating the previous finding based on the amplicon sequencing of the *minD* gene [10]. However, “Gilli-Bom-4” comprises more genetically diverse strains, which may result from different strategies by which symbionts adapt and evolve in the gut community [57]. Specifically, strain-level diversity exists in each species cluster of the core gut members, but the extent of divergence differs across bee species. Species clusters from *Snodgrassella* specific to *Bombus* and *A. cerana* are less variable than those of *A. mellifera*. However, one *Gilliamella* species from *B. terrestris* exhibited a higher level of strain diversity than the other clusters. Although observations based on a limited number of artificially reared colonies imply the link between strain diversity and host phenotypes [7], the causality of genetic divergence must be tested with more samples with clear information of the geographic distribution, host genetic background, and the local diversity of flowering plants for pollinators.

## Conclusions

In this study, we have developed a bee gut bacteria genomic database that enables strain-resolved microbial studies directly from metagenomes. We then applied it to comprehensively describe queen-worker strain transmission and strain-level dynamics in the microbiota during metamorphosis and colony development of bumblebees. The differences of the routes of microbiota transmission that is associated with their life cycles apparently affect the strain-level diversity and the functional profiles of the gut community. While we used captive-bred nests to track bacterial transmission in this cohort and experimental design, all core gut species were detected in the samples. Considering indoor-reared bumblebees has fewer opportunities to acquire microbes by foraging as wild bees, it may represent part of the overall microbiota diversity and exaggerate the low level of strain diversity and functional capacity (Fig. 5). However, either the wild host or the movement to an outdoor environment only affects the prevalence of environment-derived bacteria [58, 59]. Nevertheless, the simple yet host-restricted gut community of social bees provides a promising model to understand mechanisms of strain transmission, which is crucial to the establishment of gut community in the early life stage. Follow-up studies using gnotobiotic model with designated gut members will shed more light on this process in view of the ecological and evolutionary consequences.

## Supplementary Information

**Additional file1: Supplementary Table S1.1.** List of reference bacterial genomes for custom bee gut microbial genomic database. The genomes with blue background are used as reference genomes for the SNP calling. MAGs are shown in bold brown. NA, not available.

**Additional file 2: Supplementary Fig. S1.** Genome phylogenies and species cluster classification of the six core bacterial phylotypes *Gilliamella* **(a)**, *Snodgrassella* **(b)**, *Lactobacillus* Firm-5 **(c)**, *Bifidobacterium* (**d**), *Lactobacillus* Firm-4 (**e**), and *Apibacter* (**f**) from honeybee and bumblebee guts. Cladograms on the left of each panel are maximumlikelihood trees inferred by GTDB-tk based on the amino acid sequences of bacterial marker genes. The heatmaps represent pairwise gANI values from 75% to 100%. Values larger than 95% are highlighted in green. Color bars on the right of each panel indicate the classification of the species clusters for the MIDAS profiling. Strains from different hosts (Amel, *Apis mellifera*; Acer, *Apis cerana*; Ador, *Apis dorsata*; Aand, *Apis andreniformis*; Aflo, *Apis florea*; Bom, *Bombus* spp.; Xyl; *Xylocopa* spp.) are labeled with different colors. Twenty-eight MAGs reconstructed from metagenomes of the bumblebee guts are highlighted in blue. Two isolates of *Bifidobacterium xylocopae* XV2 and *Bifidobacterium aemilianum* XV10 from *Xylocopa violacea* were also included. **Supplementary Fig. S2.** Box plots showing the bacterial community size across the eight development stages. Absolute abundance of the bacterial community was estimated by qPCR targeting the 16S rRNA gene. Eight replicate samples were performed for each group. Statistical significance was tested using the non-parametric two-sided Wilcoxon rank sum test with Holm correction and was indicated by different letters (with P-value < 0.05 as significance threshold). **Supplementary Fig. S3.** Relative abundance of the core bacterial phylotypes shifts along the development stages of bumblebee. **(a-f)** Boxplots displayed the relative abundance of *Apibacter* **(a)**, *Gilliamella* **(b)**, *Snodgrassella* **(c)**, *Lactobacillus* Firm-5 **(d)**, *Lactobacillus* Firm-4 **(e)**, and *Bifidobacterium* **(f)** in bumblebees from different development stages. Statistical significance was tested using the non-parametric two-sided Wilcoxon rank sum test with Holm correction and was indicated by different letters (with P-value < 0.05 as significance threshold). **Supplementary Fig. S4.** Relative abundance of the six core bacterial phylotypes in the guts of workers from different stages of nest development. (a-f) Boxplots displayed the relative abundance of *Apibacter* **(a)**, *Gilliamella* **(b)**, *Snodgrassella* **(c)**, *Lactobacillus* Firm-5 **(d)**, *Lactobacillus* Firm-4 **(e)**, and *Bifidobacterium* **(f)** in the metagenomic data of the worker bee guts. Statistical significance was tested using the non-parametric twosided Wilcoxon rank sum test with Holm correction and was indicated by different letters (with *P*-value < 0.05 as significance threshold). **Supplementary Fig. S5.** Strain-level phylogenomic tree of the “Api-Bom” species cluster based on the concatenated alignments of consensus-alleles found in the core-genome. **Supplementary Fig. S6.** Relative abundance of KEGG annotations according to the KEGG Pathway categories in the metagenomes of worker bees from *B. terrestris*, *A. cerana*, and *A. mellifera*. **Supplementary Fig. S7.** Normalized gene abundance of GH23 in the gut metagenomes of bumble and honey bee species. Statistical significance was tested using the non-parametric two-sided Wilcoxon rank sum test with Holm correction and was indicated by different letters (with *P*-value < 0.05 as significance threshold).

## Data Availability

Sequencing data of the metagenomes (SRR13805672-SRR13805711) and 16S rRNA-based amplicon sequencing (SRR13790393-SRR13790540) has been deposited under BioProject PRJNA705073. The MAGs have been deposited under the GenBank, and the accession numbers are provided in Additional file 1: Supplementary Table S1. The list of analysis software and scripts generated for analysis has been deposited on GitHub at: https://github.com/QinzhiSu/Bombus/tree/master.

## Abbreviations

SNV: Single-nucleotide variant
CTAB: Cetyl trimethylammonium bromide
PCR: Polymerase chain reaction
ASV: Amplicon sequence variant
MAG: Metagenome-assembly genome
CAZymes: Carbohydrate-active enzymes
GH: Glycoside hydrolase
PL: Polysaccharide lyase
KEGG: Kyoto Encyclopedia of Genes and Genomes
KO: KEGG Orthology
