# Supplementary material for: Strain-level analysis reveals the vertical microbial transmission during the life cycle of bumblebee
Source: Microbiome. 2021 Nov 4;9:216. doi: 10.1186/s40168-021-01163-1 (PMC8567698; doi:10.1186/s40168-021-01163-1)
Supplement: Supplementary file 3 — Additional file 2: Supplementary Fig. S1. Genome phylogenies and species cluster classification of the six core bacterial phylotypes Gilliamella (a), Snodgrassella (b), Lactobacillus Firm-5 (c), Bifidobacterium (d), Lactobacillus Firm-4 (e), and Apibacter (f) from honeybee and bumblebee guts. Cladograms on the left of each panel are maximumlikelihood trees inferred by GTDB-tk based on the amino acid sequences of bacterial marker genes. The heatmaps represent pairwise gANI values from 75% to 100%. Values larger than 95% are highlighted in green. Color bars on the right of each panel indicate the classification of the species clusters for the MIDAS profiling. Strains from different hosts (Amel, Apis mellifera; Acer, Apis cerana; Ador, Apis dorsata; Aand, Apis andreniformis; Aflo, Apis florea; Bom, Bombus spp.; Xyl; Xylocopa spp.) are labeled with different colors. Twenty-eight MAGs reconstructed from metagenomes of the bumblebee guts are highlighted in blue. Two isolates of Bifidobacterium xylocopae XV2 and Bifidobacterium aemilianum XV10 from Xylocopa violacea were also included. Supplementary Fig. S2. Box plots showing the bacterial community size across the eight development stages. Absolute abundance of the bacterial community was estimated by qPCR targeting the 16S rRNA gene. Eight replicate samples were performed for each group. Statistical significance was tested using the non-parametric two-sided Wilcoxon rank sum test with Holm correction and was indicated by different letters (with P-value < 0.05 as significance threshold). Supplementary Fig. S3. Relative abundance of the core bacterial phylotypes shifts along the development stages of bumblebee. (a-f) Boxplots displayed the relative abundance of Apibacter (a), Gilliamella (b), Snodgrassella (c), Lactobacillus Firm-5 (d), Lactobacillus Firm-4 (e), and Bifidobacterium (f) in bumblebees from different development stages. Statistical significance was tested using the non-parametric two-sided Wilcoxon rank sum t [file 40168_2021_1163_MOESM3_ESM.pdf]

Supplementary Figures

Strain-level analysis reveals the vertical microbial transmission during the life cycle of bumblebee

Qinzhi Su[1,#], Qinglin Wang[1,#], Xiaohuan Mu[1], Hao Chen[2], Yujie Meng[1], Xue Zhang[3], Li Zheng[2], Xiaosong Hu[1], Yifan Zhai[2,*], Hao Zheng[1,*]

[1]College of Food Science and Nutritional Engineering, China Agricultural University, Beijing 100083, China

[2]Shandong Institute of Plant Protection, Shandong Academy of Agricultural Sciences, Jinan 250100, China

[3]Department of Entomology, College of Plant Protection, China Agricultural University, Beijing 100083, China

[#]These authors contributed equally to this work.

*Correspondence:

Hao Zheng, hao.zheng@cau.edu.cn; Yifan Zhai, zyifan@tom.com

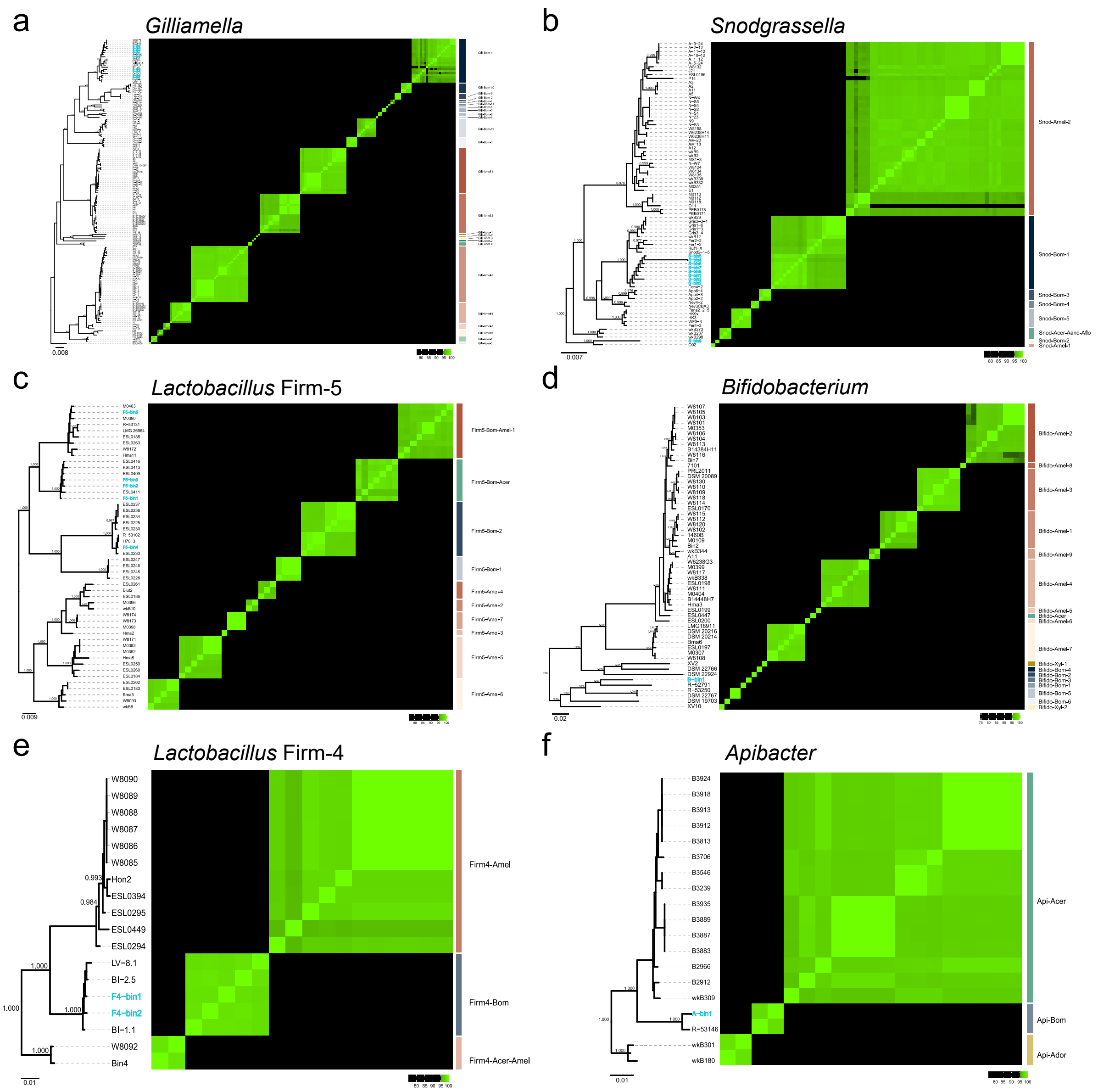

Supplementary Fig. S1. Genome phylogenies and species cluster classification of the six core bacterial phylotypes *Gilliamella* **(a)**, *Snodgrassella* **(b)**, *Lactobacillus* Firm-5 **(c)**, *Bifidobacterium* (d), *Lactobacillus* Firm-4 (e), and *Apibacter* (f) from honeybee and bumblebee guts. Cladograms on the left of each panel are maximum-likelihood trees inferred by GTDB-tk based on the amino acid sequences of bacterial marker genes. The heatmaps represent pairwise gANI values from 75% to 100%. Values larger than 95% are highlighted in green. Color bars on the right of each panel indicate the classification of the species clusters for the MIDAS profiling. Strains from different hosts (Amel, *Apis mellifera*; Acer, *Apis cerana*; Ador, *Apis dorsata*; Aand, *Apis andreniformis*; Aflo, *Apis florea*; Bom, *Bombus* spp.; Xyl; *Xylocopa* spp.) are labeled with different colors. Twenty-eight MAGs reconstructed from metagenomes of the bumblebee guts are highlighted in blue. Two isolates of *Bifidobacterium xylocopae* XV2 and *Bifidobacterium aemilianum* XV10 from *Xylocopa violacea* were also included.

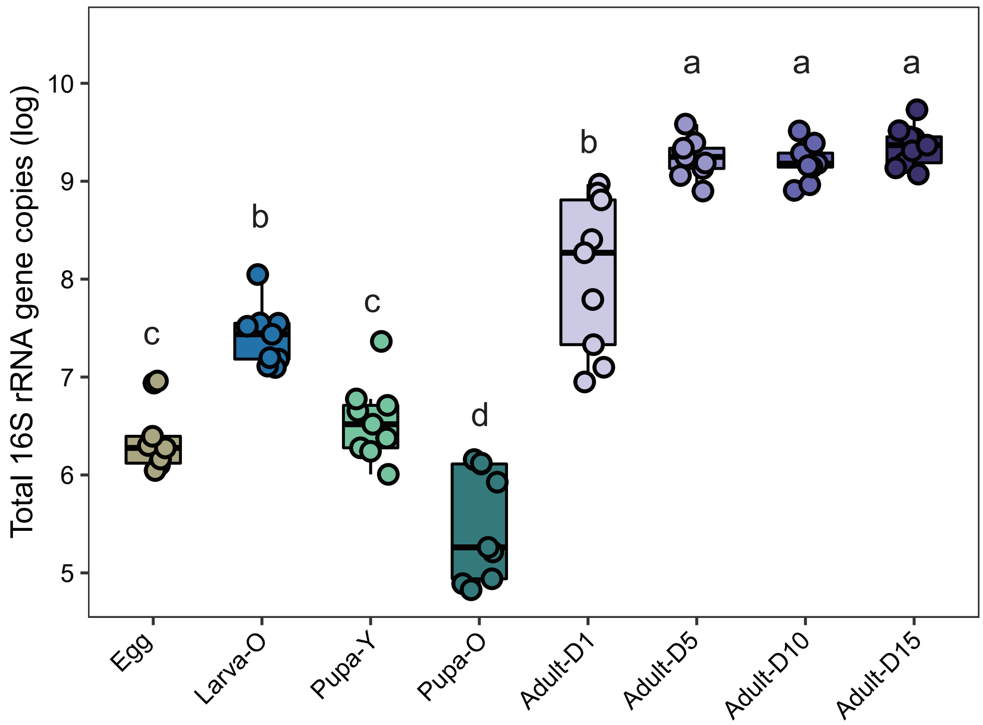

Supplementary Fig. S2. Box plots showing the bacterial community size across the eight development stages. Absolute abundance of the bacterial community was estimated by qPCR targeting the 16S rRNA gene. Eight replicate samples were performed for each group. Statistical significance was tested using the non-parametric two-sided Wilcoxon rank sum test with Holm correction and was indicated by different letters (with P-value < 0.05 as significance threshold).

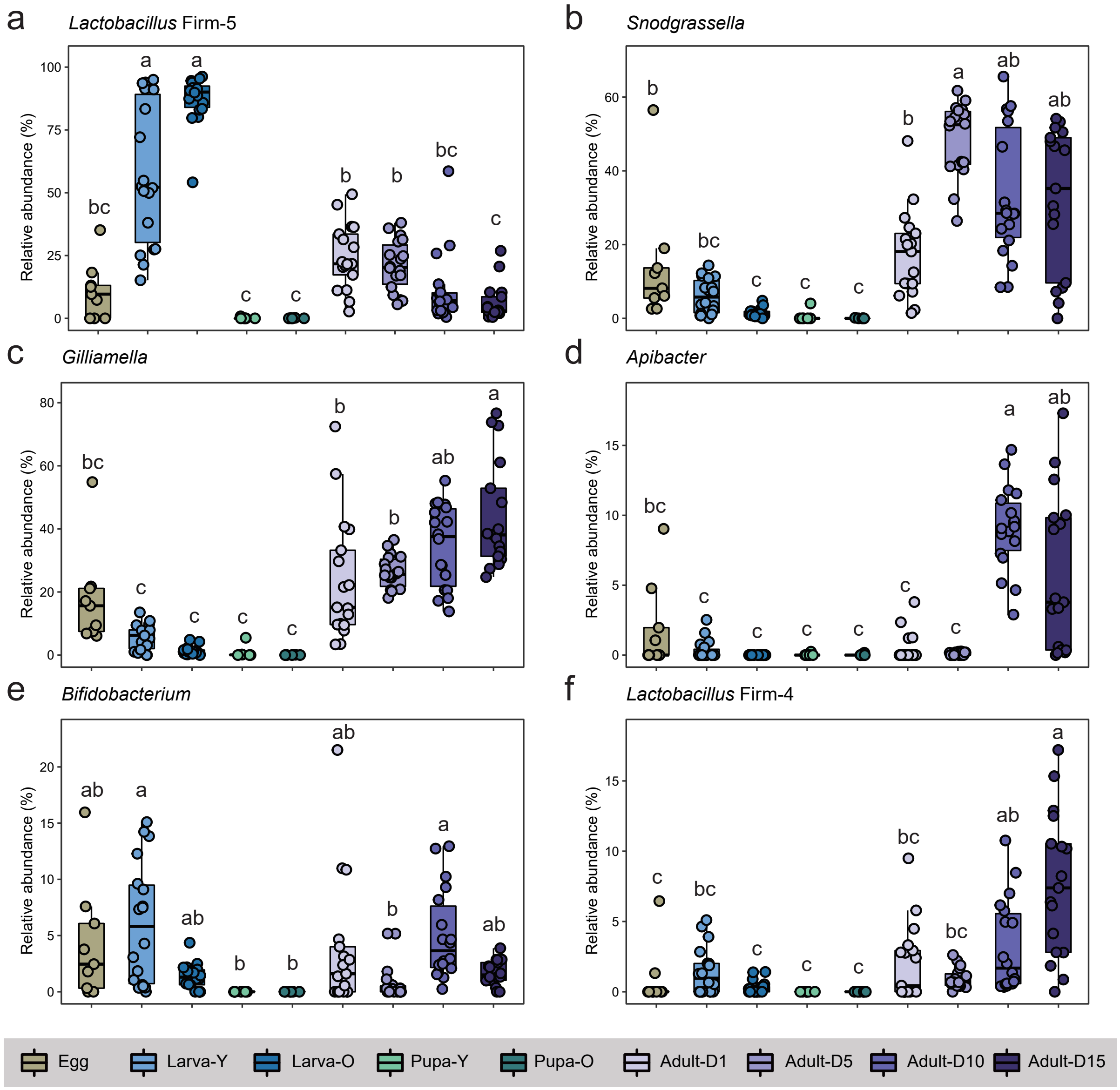

Supplementary Fig. S3. Relative abundance of the core bacterial phylotypes shifts along the development stages of bumblebee. **(a-f)** Boxplots displayed the relative abundance of *Apibacter* **(a)**, *Gilliamella* **(b)**, *Snodgrassella* **(c)**, *Lactobacillus* Firm-5 **(d)**, *Lactobacillus* Firm-4 **(e)**, and *Bifidobacterium* **(f)** in bumblebees from different development stages. Statistical significance was tested using the non-parametric two-sided Wilcoxon rank sum test with Holm correction and was indicated by different letters (with P-value < 0.05 as significance threshold).

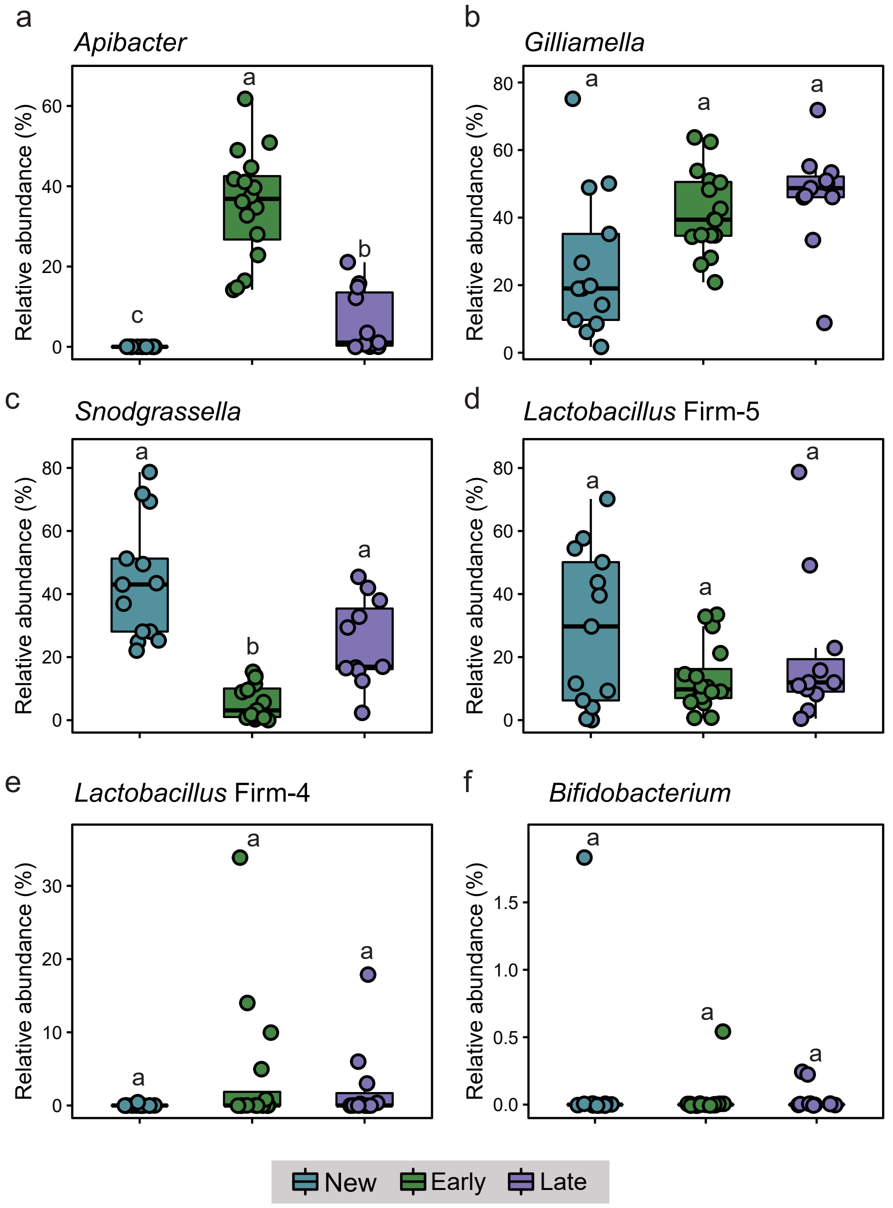

Supplementary Fig. S4. Relative abundance of the six core bacterial phylotypes in the guts of workers from different stages of nest development. (a-f) Boxplots displayed the relative abundance of *Apibacter* **(a)**, *Gilliamella* **(b)**, *Snodgrassella* **(c)**, *Lactobacillus* Firm-5 **(d)**, *Lactobacillus* Firm-4 **(e)**, and *Bifidobacterium* **(f)** in the metagenomic data of the worker bee guts. Statistical significance was tested using the non-parametric two-sided Wilcoxon rank sum test with Holm correction and was indicated by different letters (with P-value < 0.05 as significance threshold).

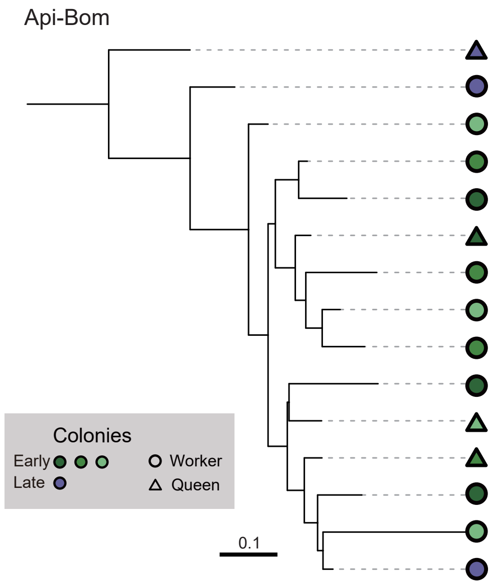

Supplementary Fig. S5. Strain-level phylogenomic tree of the “Api-Bom” species cluster based on the concatenated alignments of consensus-alleles found in the core-genome.

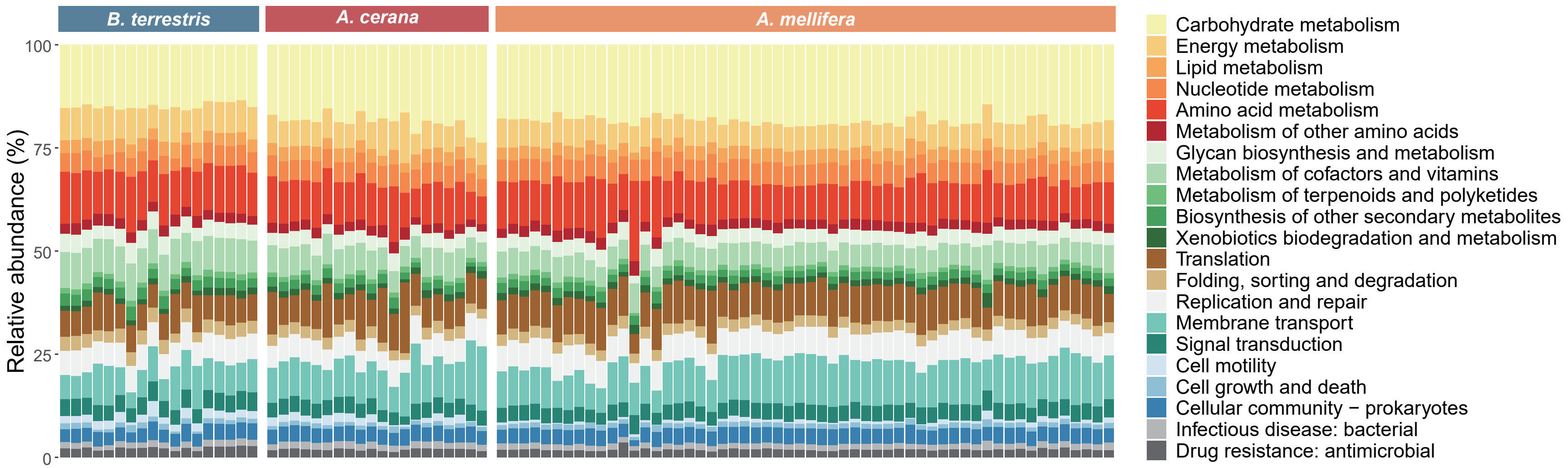

Supplementary Fig. S6. Relative abundance of KEGG annotations according to the KEGG Pathway categories in the metagenomes of worker bees from *B. terrestris*, *A. cerana*, and *A. mellifera*.

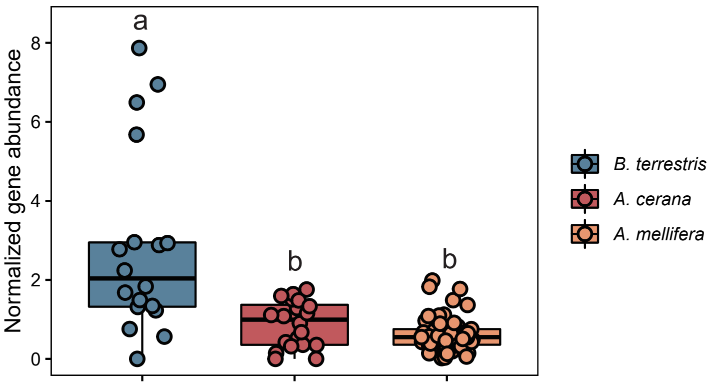

Supplementary Fig. S7. Normalized gene abundance of GH23 in the gut metagenomes of bumble and honey bee species. Statistical significance was tested using the non-parametric two-sided Wilcoxon rank sum test with Holm correction and was indicated by different letters (with P-value < 0.05 as significance threshold).
